# Oxidative modifications of tuberculosis antigen Ag85B alter its T cell antigenicity

**DOI:** 10.1016/j.redox.2026.104233

**Published:** 2026-05-28

**Authors:** Ramona Clemen, Björn Corleis, Tobias Dallenga, Paul Schulan, Ulrich E. Schaible, Sander Bekeschus

**Affiliations:** a ZIK plasmatis, Leibniz Institute for Plasma Science and Technology (INP), Greifswald, Germany; b Institute of Immunology, Friedrich-Loeffler-Institute (FLI), Greifswald, Germany; c Division of Cellular Microbiology, Program Area Infections, Research Center Borstel, Leibniz Lung Center, Borstel, Germany; d German Center for Infection Research (DZIF), Partner Site Hamburg-Lübeck-Borstel-Riems, Borstel, Germany; e Biochemical Microbiology and Immunochemistry, University of Lübeck, Lübeck, Germany; f Department of Dermatology, Venerology, and Allergology, Rostock University Medical Center, Rostock, Germany

**Keywords:** CAP, cold physical plasma, inflammation, reactive oxygen species, ROS, oxPTM

## Abstract

*Mycobacterium tuberculosis* (Mtb) remains a global health threat, necessitating innovative vaccine strategies that transcend traditional antigen delivery. While oxidative stress is a hallmark of the host-pathogen interface, the deliberate use of reactive oxygen and nitrogen species (RONS) to modulate antigen immunogenicity remains underexplored. We employed cold physical plasma, a potent source of diverse reactive species, to engineer oxidatively modified Ag85B (oxAg85B) variants. Using high-resolution mass spectrometry, we performed comprehensive oxidative protein modification mapping, identifying a landscape of 59 distinct oxPTMs. The functional impact on cellular immunity was evaluated using transgenic mice harboring Ag85B-specific CD4^+^ T-cells. Gas plasma treatment significantly enhanced the immunogenic profile of Ag85B. Compared to native controls, oxAg85B potentiated CD4^+^ T-cell activation and increased interferon-gamma secretion in an antigen-dependent manner. Systematic correlation analysis revealed that the "redox fingerprint" of the antigen was strictly dependent on the plasma operation mode. Notably, hydroxyl radical-rich plasma environments favored a pro-inflammatory Th17-linked profile. High-dimensional mapping showed that elevated IL-17α release strongly correlated with a specific cluster of modifications, including dihydroxylation, deamidation, and protein carbonylation. Crucially, vaccination with oxAg85B increased systemic inflammatory cytokines without altering anti-Ag85B antibody titers, suggesting a selective enhancement of cell-mediated immunity without compromising B-cell priming.

## 1 Introduction

Tuberculosis (TB) is a common and often deadly infectious disease, and rising drug resistance is a further challenge and burden for the healthcare system. TB is caused by members of the *Mycobacterium (M.) tuberculosis* complex, mainly *M. tuberculosis* (Mtb.), or including *M. bovis* [1], the causative agent of cattle TB. The latter provided the basis to generate the attenuated Bacillus Calmette-Guérin (BCG) vaccine strain, which is currently the only available and widely used vaccine against TB. BCG is not routinely given in Europe or the United States due to its low efficacy against TB. However, it is still used in many other countries to protect infants against severe forms of TB meningitis [2]. However, immunization with BCG has been shown to protect against a primary infection with *M. tuberculosis*, but its protective efficacy against TB reactivation, particularly pulmonary TB, is not satisfactory. Increasing resistance mechanisms make the bacteria less susceptible to treatments, which poses a global health challenge, underscoring the need for novel and innovative preventive strategies against active TB [3].

Within granulomas of active TB patients, host responses are associated with oxidative conditions through NOX2 and MPO-generated reactive oxygen intermediates primarily during the interaction of neutrophils with the pathogen. While this interaction not only fails to control mycobacterial growth, it also promotes lung tissue destruction, resulting in TB disease exacerbation, pathogen transmission, and post-TB disease. Moreover, the mycobacteria become exposed to oxidative stress, and mycobacterial antigens, especially those surface exposed or secreted, can become oxidized with a putative effect on their immunogenicity.

There is current interest in investigating the defense mechanisms of Mycobacteria against reactive oxygen and nitrogen species (ROS) [4,5]. For instance, ROS and ROS-induced modifications on biomolecules may act as natural adjuvants, enhancing existing adaptive immunity. To resist cytotoxic ROS effects and minimize redox-stress-mediated cell damage, microbial pathogens need to adapt, and other mechanisms have also been observed in mycobacteria. For example, Galagoda and colleagues found altered gene profiles in chlorine-treated Mycobacteria that indicate a multifaceted adaptation to oxidative stress due to detoxification (nemA), redox homeostasis (COQ5; rosB), protein homeostasis (cysHKO, moeZ), and different lipid metabolism (cpnA) [6].

Investigating the effect of ROS during phagocytosis, Parker and colleagues found the resistance of nontuberculous mycobacteria (NTM) *M. smegmatis* to hypochlorous acid (HOCl) mediated phagosomal killing, suggesting sensitivity to superoxide, which is generated by neutrophils [7]. Indeed, NADPH-dependent phagocyte oxidase (Phox) complex produces antimicrobial superoxide and is a major regulator for *Mtb* infection [8]. For neutralizing superoxide, *Mtb* produces the antioxidant enzyme superoxide dismutase (SOD), and also other bacteria express SOD to resist superoxide during oxidative burst and killing, such as *S. aureus* [9]. In *Mtb*, SOD was shown to be required for resistance to killing by exogenously generated superoxide [10]. Furthermore, ROS treatment triggered the expression of genes essential for antioxidant adaptation and protection processes in *M. bovis* [11] and *Mtb* [12]. During oxidative burst (e.g., in neutrophils), the cell wall of tuberculosis is primarily affected, which includes the mycobacterial enzymes mycolyl transferase antigens (Ag)85A, Ag85B, and Ag85C. These enzymes are essential for lipid transfer in the protective mycomembrane, and bactericidal reagents target these enzymes to reduce growth [13]. Crystal structure analysis identified the amino acids Serin at position 148 and Histidin at position 260 as being essential for structural changes in Ag85C and acyltransferase activity [14]. Conversely, Mandato and Chai proposed oxidative post-translational modification of the cysteine residue in Ag85C to decrease enzyme activity, as the modification induced by biotinylated glutathione ethyl ester was rescued by adding dithiothreitol [15].

Although it becomes increasingly clear that oxidative protein modifications may have functional consequences in various pathophysiological conditions, little is known about their role in antigen presentation and T cell activation in the context of TB. Here, we introduced several oxidative modifications to the tuberculosis antigen Ag85B, using a technology generating hydroxyl radicals, superoxide, singlet oxygen, and several others at room temperature [16,17]. We then used oxidized Ag85B to study antigen-specific T cell responses in vitro, as well as animal vaccination to investigate antibody production and secretion profiles in vivo. Finally, specific protein modifications were correlated against functional responses in immune cells to identify potential drivers of these effects. As Ag85B, due to its T cell antigenicity, is a principal component of two subunits and one vector-based vaccine candidates currently in clinical testing [18]. Our findings may therefore contribute to the further development of a more immunogenic split vaccine against TB.

## 2 Results

### 2.1 Oxidized Ag85 was not toxic and modulated antigen-specific T cell responses

**Fig. 1:**
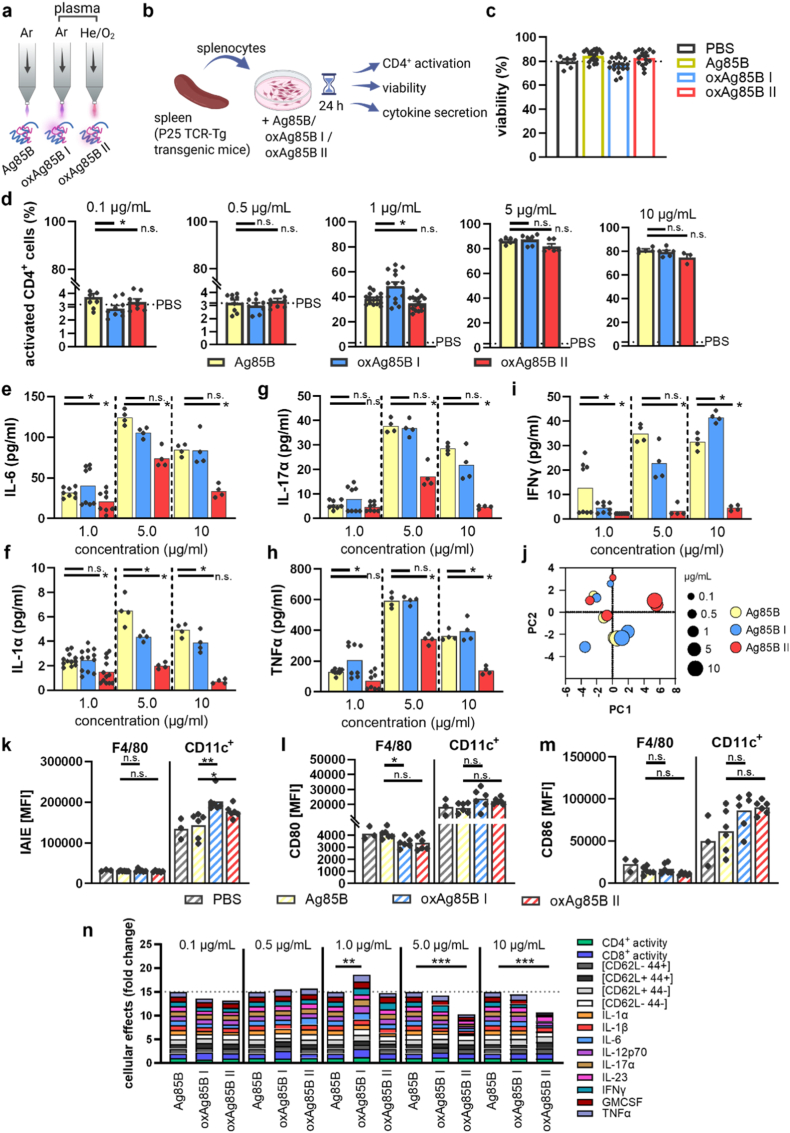
**Oxidized Ag85B increased CD4^+^ activity in splenocytes from p25 animals.** Splenocytes from transgenic P25 animals were isolated and incubated with Ag85B, argon gas plasma-treated Ag85B (oxAg85 I), or helium-oxygen gas plasma-treated Ag85B (oxAg85B II), respectively, prior to determining cellular response by flow cytometry and multiplex assay. (**a**) Scheme of gas plasma treatment; (**b**) experimental design; (**c**) viability after 24h incubation with Ag85B, or oxAg85B variants (**d**) and T cell activation (CD69^+^/CD25^+^); (**e-h**) secretion analysis in supernatants harvested at 24h for (**e**) IL-6, (**f**) IL-1α, (**g**) IL-17α, (**h**) TNFα and (**i**) IFNγ; (**j**) overall cellular responses to Ag85B and oxAg85B at different concentrations visualized via principle component analysis, considering activation, subpopulations, CD8^+^ activation (see Fig. S1), and secretion profiles; spenocytes from C57BL6 mice were isolated and incubated with PBS, Ag85B or oxAg85B variants for 24h. Antigen-presenting cells were scratched, and activation markers were detected by flow cytometry (**k**) IAIE; (**l**) CD80; (**m**) CD86. (**n**) Summarized fold change of individual cellular response in splenocytes from p25 animals to Ag85 and oxAg85B. Data are mean (**c, d, k-m**) from three to five independent experiments with two to three technical replicates each, with statistical analysis performed using Mann-Whitney test (oxAg85B vs. Ag85B). Data are mean ± SEM (**e-h**) from quadruplicates of pooled samples from three biological replicates with three technical replicates each, with statistical analysis performed using the Mann-Whitney test (oxAg85B vs. Ag85B).∗ = p < 0.05, ∗∗ = p < 0.01, ∗∗∗ = p < 0.001, n. s. = not significant.

We previously tested an antigen-specific T cell response to gas plasma-treated ovalbumin in splenocytes from OT-II mice and found different activation rates when using two different gas compositions [19]. We therefore used the same feed gas conditions to treat the immunogenic protein Ag85B of *Mycobacterium tuberculosis* in PBS, namely argon to generate oxAg85B I and helium oxygen for oxAg85 II; argon gas was used as a control (Fig. 1a). The antigen specific CD4^+^ T cell response was elucidated in splenocytes isolated from P25 transgenic TCR animals (specific to Ag85B [20]) by determining activation rate, proliferation and cytokine secretion (Fig. 1b). Importantly, neither Ag85, nor oxidized variants were toxic for splenocytes (Fig. 1c). Flow cytometry analysis revealed a threshold activation for antigen-specific T cells at a concentration between 0.5 μg/ml and 1 μg/ml and increasing concentrations elevated T cell activity until a maximum activity of ∼80% (Fig. 1d–S1a-b). An increased percentage of CD69^+^/CD25^+^ double-positive CD4^+^ T cells was found in splenocytes after incubation with oxAg85B I at concentrations of 1 μg/ml and 5 μg/ml, but not with oxAg85 II. Oxidized antigen at the highest concentration (10 μg/ml) did not further increase T cell activity. Although P25 transgenic mice have a transgenic CD4^+^ T cell population and naïve or natural CD8^+^ T cell repertoire, we found an increased percentage of activated CD8^+^ T cells after incubation with oxAg85B I (Fig. S1c). We further isolated immune cells from the thymus, and there were no changes observed in CD3^+^ lymphocyte activation after incubation with oxidized Ag85B (Fig. S1d).

In previous studies, we detected a shift in the activation rate of different T cell subpopulations after incubation with gas plasma-oxidized ovalbumin, MART, and PMEL [19,21]. Herein, activated T cells showed increased memory (T_cm_) or effector (T_eff_) phenotype. Still, higher concentrations of Ag85B and oxidized Ag85B variants reduced the percentage of activated CD62L^−^CD44^+^ cells (Fig. S1e). On the other hand, oxidized Ag85B I and II did not lead to a shift in percentage subpopulations when compared to Ag85B. As immune cell activation often correlates with cytokine secretion, we further determined cytokine concentrations in the supernatants via multiplex assay. Interleukin (IL)-6, IL-12, GM-CSF, and tumor necrosis factor (TNF) α showed increased levels after incubation with 1 μg/ml oxAg85B I when compared to native Ag85B (Fig. 1e–i, S2a-f). On the other hand, the pro-inflammatory cytokines IL-6, IL-17a, IL-1α, interferon (IFN)γ, and TNFα were significantly reduced after incubation with oxAg85 II at all concentrations when compared to Ag85B. INFγ- and IL-1α also showed significant reduction in response to oxAg85B I at 1 and 5 μg/ml, or 5 and 10 μg/ml, respectively. Low concentrations of Ag85B or oxidized variants did not lead to significant changes, except TNFα, which was reduced in oxAg85B condition (Fig. S2g–h). Considering the activation rate, percentage of subpopulations, cytokine levels, and proliferation, we visualized the differences by principal component analysis (Fig. 1j–S1f). Low concentrations of Ag85B and oxAg85B variants clustered together due to the activation threshold rate, and the effects of high oxAg85B II were separated from Ag85B and oxAg85B I.

**Fig. 2:**
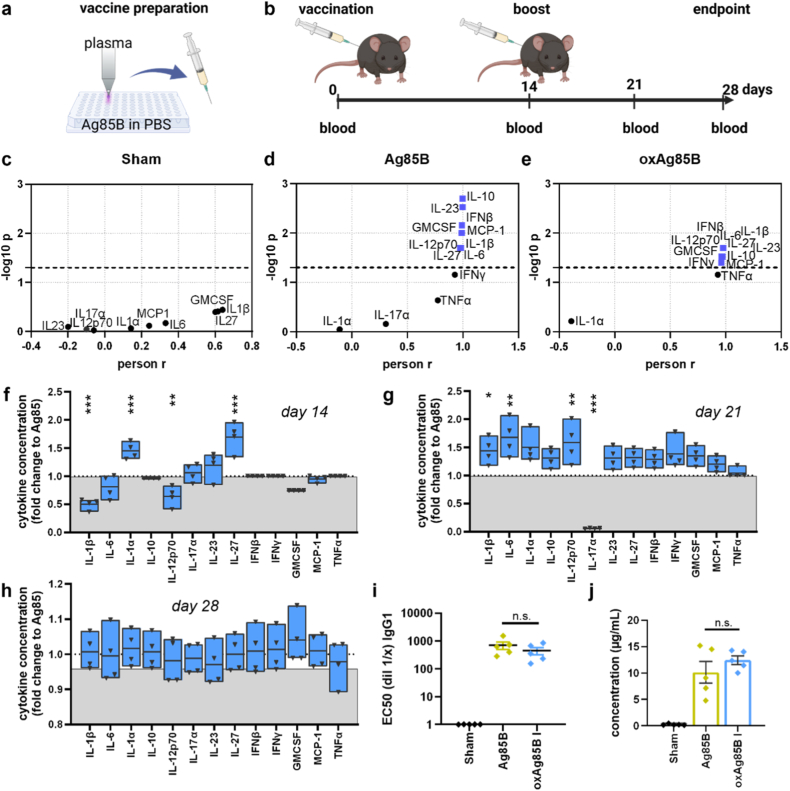
**Immunization with oxAg85B, blood plasma secretion profiling, and Ag85B antibody levels.** (**a**) Scheme of vaccine preparation; (**b**) B16F10 wild-type animals immunized with Ag85B, argon gas plasma-treated Ag85B (oxAg85B I), or sham (PBS), and blood collection at days 0, 14, 21, and 28; (**c-e**) correlation analysis of blood serum analytes against collection day for sham (**c**), Ag85B (**d**), and oxAg85B I (**e**); (**f-h**) cytokine levels in blood of oxAg85B I vaccinated animals normalized to native Ag85B vaccinated animals after 14 days (**f**), 21 days (**g**), and 28 days (**h**); determination of antibody levels reactive against native (**i**) or oxidized Ag85B (**j**) at day 28 from animals previously vaccinated native or oxidized Ag85B. Data are mean from five vaccinated animals per group with statistical analysis performed using Pearson correlation (blue values = p < 0.05) (**c-e**) or Mann-Whitney test (**f-i**) (oxAg85B vs. Ag85B). ∗ = p < 0.05, ∗∗ = p < 0.01, ∗∗∗ = p < 0.001, n. s. = not significant.

T cell activation is mediated via antigens presented on the MHC complex of antigen-presenting cells. Splenocytes of native C57BL6 mice were incubated with Ag85B or oxAg85B variants for 24h, and activation of myeloid cells and T cells was determined via flow cytometry. Herein, MHC-II was increased on CD11c^+^ cells after incubation with 1 μg/ml oxAg85 I, but oxAg85B II reduced MHC-II on macrophages (F4/80) and dendritic cells (CD11c^+^) (Fig. 1k). On the contrary, activation and co-stimulation marker CD80 was reduced, and CD86 was not affected after incubation with oxAg85B variants (Fig. 1l and m). Importantly, activation of CD4^+^ T cells from wild-type mice was not affected, whereas an increased number of activated CD8^+^ T cells was determined after incubation with oxAg85B I (Fig. S1f). With a focus on antigen-specific CD4^+^ T cells, notably, 1 μg/ml oxAg85B I was separated from all other conditions when comparing different concentrations. Since argon gas plasma mainly produces hydroxyl radicals (and, as a more long-lived agent, hydrogen peroxide (H_2_O_2_)), we treated Ag85B with equimolar H_2_O_2_ concentrations. Still, we did not see any increase in T cell activation when compared to Ag85B (Fig. S1h–j). Summarizing cytokine secretion and T cell activation in splenocytes from p25 animals after incubation with oxAg85B variants, a significant increase was observed for argon gas plasma-treated Ag85B when compared to Ag85B, whereas oxAg85B II led to a reduced effect (Fig. 1k). We therefore used oxAg85B I for further experiments in vivo (Fig. 2).

### 2.2 Vaccination with oxidized Ag85B left native Ag85B-antibody production intact

The gold standard method to investigate the immunogenicity of biomolecules is in vivo immunization prior to immune response analysis. In our experiments, naïve C57BL6 mice received a prime (day 0) and a boost (day 14) injection with native or oxAg85B, or vehicle (sham) (Fig. 2a–b). The immune response was elucidated by detecting cytokines and antibodies in serum and T cell activity after restimulation ex vivo. Interestingly, a correlation of the cytokine release measures over different time points revealed no significant increase in the control animals, but in serum from animals vaccinated with Ag85 or oxAg85 (Fig. 2c–e, S3a). Concentrations of GMCSF, IL-1β, IL-6, IL-10, IL-12p70, IL-23, IL-27, IFNβ, and MCP-1 were significantly increased over time after Ag85B and oxAg85B vaccination. Additionally, increased levels of IFNγ and IL-17a were detected in the serum of oxAg85B vaccinated animals over time. We further compared the serum cytokine levels from animals after vaccination with Ag85 and oxAg85B for the individual time points and found elevated levels of IL-1α and IL-27, and reduced IL-1β and IL-12p70 concentrations in oxAg85B vaccinated animals after 14 days (Fig. 2f). Seven days after boost injection, IL-1β, IL-12p70, and IL6 were significantly increased in serum of oxAg85B-vaccinated animals (Fig. 2g). Albeit IL-17a concentrations were generally rising over time in oxAg85B-vaccinated animals, it was in an overall lower concentration when compared to Ag85B-vaccinated mice. (Fig. S3b). Two weeks after boost vaccination, serum cytokine levels were similar in Ag85B and oxAg85B-vaccinated animals (Fig. 2h). We did not detect changes in the antibody level targeting native Ag85B (Fig. 2i–S3b). Antibodies targeting oxAg85B were slightly, but not significantly, upregulated in oxAg85B vaccinated animals (Fig. 2i). Spleens from vaccinated animals were taken to re-stimulate immune cells with Ag85B peptide or activation cocktail in vitro prior to flow cytometry analysis. However, we did not detect significant changes in CD4^+^ or CD8^+^ T cell activation, measured by intracellular perforin, IFN-γ, or IL-17 (Fig. S3c–f). In summary, vaccination in vivo revealed that oxAg85B I did not modulate the immunization response to native Ag85B but altered the immune response at early time points. Suggesting that oxidative modifications modify the immunization process, we were interested in oxidative modifications on amino acids in Ag85B after exposure to gas plasma.

### 2.3 Hydroxylation patterns were enriched in oxAg85B I

**Fig. 3:**
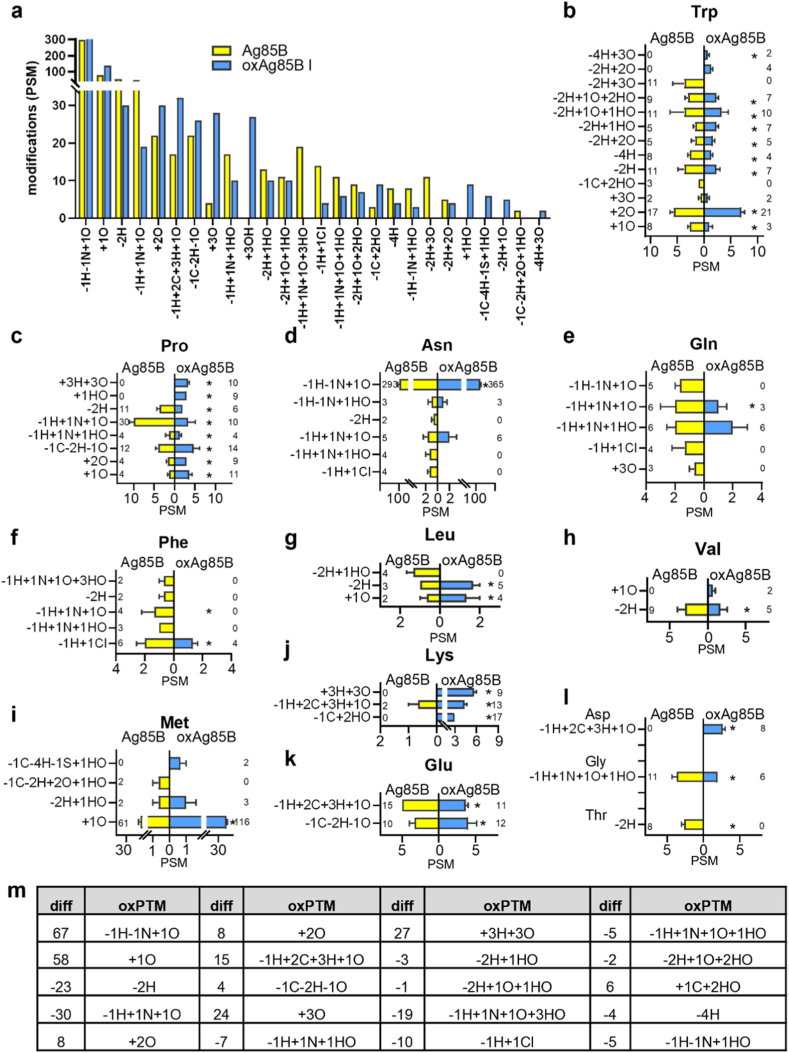
**Gas plasma treatment induced several Ag85B modifications, mainly hydroxylation.** (**a**) Ag85B and argon gas plasma-treated Ag85B (oxAg85 I) were digested, acetylated, and reduced prior to mass spectrometry, and amino acid sequence and accumulated oxPTM detected in Ag85B and oxAg85B are shown; (**a**) detected peptide spectrum matches (PSM) for specific oxidative modifications; (**b-l**) oxPTMs (mean PSM) on individual amino acids in oxAg85B compared to Ag85B and total PSM count; (**m**) 20 most common oxPTMs identified by highest PSM numbers and differences in oxAg85B.

Oxidative modifications on Ag85B and oxAg85B I were investigated using high-resolution mass spectrometry to correlate changes with cytokine production in vivo. Various mass shifts for oxidative modifications were identified on different amino acids in Ag85B and oxAg85B (Fig. 3a, S4a, Table S1). Some were specifically found after plasma exposure, such as hydroxylation (+1OH, +3OH, -2H+1O). Most changes in PSMs comparing Ag85B and oxAg85 I were found on Trp, including -2H+2O, having a total count of 4 in three replicates (Fig. 3b). Furthermore, +2O was significantly increased after plasma exposure (total PSM of 21 in oxAg85B vs. 17 in Ag85B). The increased PSMs on Pro included various oxPTMs, such as hydroxylation (+1OH, +3OH) (Fig. 3c). On the contrary, Asn, Gln, and Phe showed decreased oxidative modifications after exposure to gas plasma (Fig. 3d–f). Other amino acids showed fewer changes (Fig. 3g–l) or no oxPTMs at all, such as Ala and Cys (**Data not shown**). In general, hydroxylation alone or in combination was the most prominent modification, which was increased in many amino acids after exposure to gas plasma (Fig. 3m). The overall most common modification on oxAg85B was a deamidation (-1H–1N+1O) with more than 200 PSM, which was mainly detected on Asn, Gln (Fig. 3d and e). Importantly, in oxAg85B, there were more (67) deamidations found when compared to Ag85B (Fig. 3m). Other common oxPTMs, out of the 20 most common oxPTMs, showed increased PSM in oxAg85B I, such as +1O (58 more), or were decreased, such as -1H+1 N+1O (30 less). Interestingly, +1O, +2O and +3O were increased in oxAg85B. The question arose whether those modifications correlate with cellular response detected in vitro and in vivo.

### 2.4 Functional immune cell alterations significantly correlated with Ag85B I oxPTMs

**Fig. 4:**
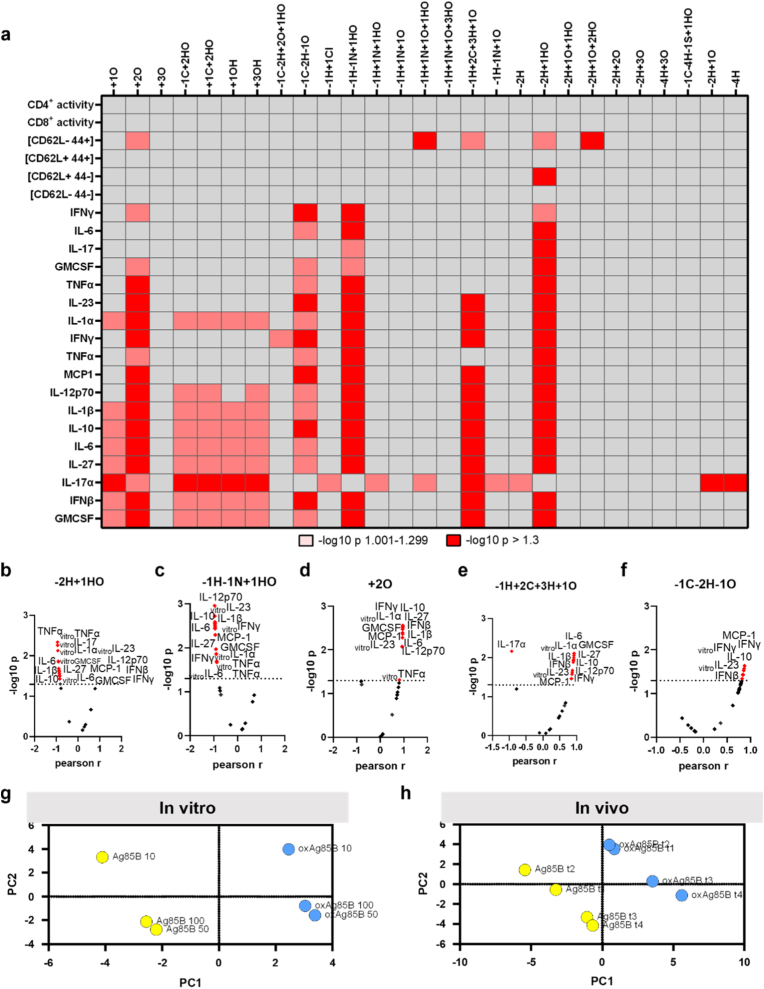
**Hydroxylation correlated with immune cell activity.** (**a-f**) Pearson correlation of Ag85B modifications correlated against in vitro and in vivo secretion and cell activation, significant p values in red and positive or negative correlation shown for (**b**) -2H+1HO, (**c**) -1H–1N+1HO, (**d**) +2O, (**e**) -1H+2C+3H+1O, (**f**) -1C–2H–1O; (**g-h**) PCA conducted for cumulated modifications stratified for in vitro (**g**) or in vivo (**h**) results.

We further correlated oxPTMs with biological response of the in vitro and in vivo results, performing Pearson correlation (Fig. 4a, S5). Hydroxylation was the overall most common modification that significantly correlated with different biological markers (Fig. 4a). For instance, -2H+1OH negatively correlated with 17 out of 24 markers (Fig. 4b), Deamidation (-1H–1N+1OH) negatively correlated with 15 cytokines (Fig. 4c). On the contrary, dioxidation (+2O) positively correlated with ten cytokines in vitro and in vivo (Fig. 4d), and -1H+2C+3H+1O positively correlated with 12 cytokines and negatively with IL-17α (Fig. 4e). -1C–2H–1O showed significant biological changes with six cytokines (Fig. 4f). Interestingly, two out of the five mentioned modifications correlated with IL-17α, but seven other modifications correlated with IL-17α and no other marker (Fig. S5). Herein, seven modifications negatively correlated with IL-17α, and two modifications (-4H and -1C+2OH) positively correlated with the cytokine secretion. Comparing the 11 most abundant modifications (-1H–1N+1O, +1O, -2H, -1H+1 N+1O, +2O, -1H+2C+3H+1O, -1C–2H–1O, +3O, -1H+1 N+1HO, +3H+3O) and the overall response to Ag85B and oxAg85B I, principal component analysis was conducted. The samples of the two groups were distributed, and no clustering was observed for in vitro results, but there was a clustering within the groups of 50 μg/ml and 100 μg/ml in vitro (Fig. 4g). In vivo, PCA considering oxPTMs and cytokine levels at different time points showed a clustering of t1 and t2 in oxAg85B I condition and t3 and t4 in Ag85B (Fig. 4h). Interestingly, effects of different time points (in vivo) and concentrations (in vitro) distinguished based on cytokine secretion (PC2), whereas the main differences between Ag85B and oxAg85B was based on hydroxylation (PC1) (Fig. S4b and S4c). All in all, we see a significant effect of hydroxylation motifs on immunogenic effects.

## 3 Discussion

Mtb is recognized by innate immune cells via pattern recognition receptors (PRRs), including Toll-like receptors (TLRs), and initiates cytokine secretion for immune cell recruitment (reviewed in Ref. [22]). During the host's first-line defense, superoxide and hypochlorous acid are produced to eliminate the bacteria and may induce oxidative posttranslational modification to promote immune cell recognition. After phagocytosis, proteins are digested, and peptides are presented on the cell surface via major histocompatibility complex (MHC) to initiate an adaptive immune response. This involves activation of antigen-specific CD4^+^ T cells, CD8^+^ T cells, and B cells, specific antibody production, and secretion of cytotoxic granules (reviewed in Ref. [23]). We here found evidence for an altered immunogenicity of oxidized Ag85 protein when compared to native Ag85B. Importantly, we did not investigate a re-challenge with *M. tuberculosis* infection after immunization, as our objective is not to develop a protective vaccine, but we found altered immunological consequences. This included stimulation of antigen-specific T cell activation in vitro and immunization process in wild-type mice in vivo, as well as antibody production by B cells in vivo. We therefore suggest a vital role for oxidized antigens and immune cell priming, which supports previous findings. With the focus on promoting an anti-tumor immune response, we previously oxidized tumor-associated antigens and whole tumor lysates with cold-physical plasma to prove if vaccination protects from tumor growth [19,21,24]. In the context of infectious diseases, proof for the altered immunogenicity of oxidized targets was missing. Here, we found differences in the activation of T cells in a transgenic mouse model and altered cytokine concentrations in the blood of immunized animals.

Antigen sensing via TLR promotes the secretion of the inflammatory cytokine IL-12, which is necessary for priming T cells. Consequently, primed T cells produce IFNγ and TNFα that are crucial for control of infection (reviewed in Ref. [22]). We here observed elevated IFNγ and TNFα secretion after incubation with 10 μg/ml (f.c.) oxAg85B, which, however, did not lead to activation of antigen-specific T cells in vitro. On the other hand, 1 and 5 μg/ml elevated T cell activation, but reduced IFNγ secretion, and led to inconsistent TNFα levels. We therefore propose that other immune cells secrete IFNγ and TNFα, and that there is antigen-unspecific activation in vitro. Other observations support this hypothesis: 1. There is increased CD8^+^ T cell activation, including at antigen concentrations below the expected threshold, although transgenic mice should not bear CD8^+^ with transgenic Ag85B-specific TCR; 2. CD4^+^ T cell response is inconsistent, as the groups of 5 μg/ml and 10 μg/ml oxAg85B clustered in the PCA, but T cell activity was stimulated with 1 μg/ml and 5 μg/ml, and not 10 μg/ml 3. There was no antigen-specific response in vivo detected, as the antibody level targeting Ag85 did not change, and no antigen-specific T cell response was determined; 4. CD8^+^ T cells from wild-type mice get activated after incubation with oxidized Ag85B Protein.

Proposing adjuvanticity, we suggest an alteration of the signaling cascade in APCs, as activation markers CD80 and MHC-II changed in macrophages and dendritic cells. Interestingly, we previously found oxidized pathogenic antigens to alter APC activation [25], and the oxidized variant of ovalbumin promoted antigen uptake [19]. In line with our findings, modified Ag85, namely Ag85A-fusion proteins, was previously shown to elevate macrophage phagocytosis [26]. Further research needs to be done to prove individual uptake mechanisms and signaling cascades in response to oxidized antigens.

To shed light on the effects of reactive species on the tuberculosis antigen Ag85B, we here investigated oxPTMs after exposure to a multi-ROS environment and linked them to biological consequences. Notably, argon gas plasma is known to produce – besides a range of short-lived reactive species [27] – hydrogen peroxide (H_2_O_2_). Adding H_2_O_2_ alone to Ag85 could not recapitulate the effects seen with gas plasma-treated Ag85, which is in line with previous reports [19,28]. We previously proposed quinone modification (-2H+3O) to be highly immunogenic, as gas plasma-induced quinone modification on oxOva and oxPMEL promoted anti-tumor immune response [19,21]. We here find reduced quinone modification on Trp in oxAg85B and no immunogenic potential, which supports our hypothesis. On the other hand, hydroxylation was not determined in the previous studies, but we here find significantly high numbers of various hydroxylation subtypes. For instance, hydroxylation (-2H+1OH) correlated with 17 of 24 markers but was mainly found in native Ag85B. We therefore suggest that this modification is not an oxidation pattern-induced immunogenic target; more often described oxPTMs in diseases are nitration (-1H+1 N+2O) [29,30], nitrosylation (-1H+1 N+1O) [31], carbamylation (+1C+1H+1 N+1O) [32], and deamidation (-1H–1N+1O) [33]. In our study, nitrosylation did not correlate with any marker; carbamylation and nitration did not appear, and deamidation (-1H–1N+1O) correlated with 15 markers. On the other hand, most oxidation patterns (8 of 27) correlated with IL17α, including modifications specifically found on oxAg85B (+3OH, +1OH, -2H+1O). IL-17-secreting T helper subsets are important inflammation mediators and promote cytotoxic T cell response [34], and we found various hydroxylation patterns, which correlated with Serum IL-17α in vivo. On the other hand, there was no increased IL-17α observed in vitro, although T cell activity was increased. These findings are in line with our previous findings, where oxOva II elevated IL-17α in OT-II mice in vivo, but not in supernatants of stimulated splenocytes [19].

Considering the complexity of an immune response in an organism, we hypothesize an altered signaling route by oxPTM in vitro and in vivo, which may depend on IL-17α. In vivo, we did not detect changes in Ag85B-specific antibody response. No changes in antibody production are a positive result from another point of view, namely, the stability of a potential protein vaccine. During the manufacturing process of monoclonal antibodies, posttranslational modifications can occur, resulting in instability and less efficacy [35]. Here, we show several oxPTMs on Ag85B that did not affect the immunization process in vivo, as Ag85B antibody production and antigen-specific T cell activation did not change. Conversely, we observed that oxAg85B led to an alternative cytokine profile at early time points, but less IL-17, suggesting an altered inflammatory response after shortened vaccination time. A promoted immunization process would positively impact the vaccination scheme and give the opportunity for a fast-track vaccine. However, more research needs to be done to determine a faster immunization process by oxAg85B vaccine, which reduces tuberculosis-associated pathology. In summary, we here show for the first time that gas plasma-induced oxidative modifications on Ag85B significantly correlate with altered cytokine secretion, but do not modulate T cell and B cell response in vivo.

## 4 Conclusion

Gas plasma-derived reactive species deliberately oxidized the Mtb antigen Ag85B. The protein modifications, including hydroxylation patterns and deamidation, significantly correlated with cytokine secretion. Furthermore, IL-17α secretion in vivo correlated with hydroxylation, deamidation, and carbamylation. Our study provides testable hypotheses in other immunological conditions in infection and beyond, further linking redox biology to specific disease characterization and novel potential routes of remission.

## Funding

This work was funded by the German Federal Ministry of Education and Research (BMBF; today, German Federal Ministry of Science, Technology, and Space, BMFTR), grant numbers 03Z22DN11 (to S.B.), 01KI2135A (to S.B.), and 03Z22Di1 (to S.B.). The funding source had no role in the design of this study or its execution, analyses, interpretation of the data, or decision to publish results.

## CRediT authorship contribution statement

**Ramona Clemen:** Conceptualization, Data curation, Formal analysis, Investigation, Methodology, Project administration, Validation, Visualization, Writing – original draft, Writing – review & editing. **Björn Corleis:** Conceptualization, Funding acquisition, Investigation, Methodology, Resources, Writing – review & editing. **Tobias Dallenga:** Conceptualization, Methodology, Resources, Writing – review & editing. **Paul Schulan:** Data curation, Formal analysis, Investigation, Methodology, Validation, Writing – review & editing. **Ulrich E. Schaible:** Conceptualization, Funding acquisition, Resources, Writing – review & editing. **Sander Bekeschus:** Conceptualization, Funding acquisition, Methodology, Resources, Supervision, Writing – review & editing.

## Declaration of competing interest

The authors declare no conflict of interest with regard to the publication of this article.

## Data Availability

Data will be made available on request.

## Experimental section

### Ag85 gas plasma exposure

Ag85 was reconstituted following the manufacturer's instructions and diluted in phosphate-buffered saline (PBS). Reactive species exposure was performed using a kINPen atmospheric pressure argon plasma jet (neoplas, Germany). Protein suspensions (100 μl per well) were added to round-bottom 96-well plates (Sarsted, Germany) and exposed to gas plasma for 10 s. Pure argon gas or helium gas containing 2% oxygen (all Air Liquide, France; purity 99.999%) was used as carrier gas, and the flow rate was set to one standard liter per minute using a high-precision flow controller (MKS, Germany). The distance between the gas plasma jet's nozzle and the liquid was >2 mm to achieve a conductive effect for maximizing reactive species transport into the liquid target containing the protein [27]. As control condition, native protein was treated with gas only (without ionization). After gas plasma treatment, the evaporated volume was compensated for by adding a predetermined amount of ddH_2_O.

### Reactive species detection

PBS was treated with plasma as described above. Nitrite and nitrate were determined by performing the Griess-assay (Cayman Chemical, Germany) as previously outlined [36], and absorbance was measured at *λ*_ex_ 548 nm. Hydrogen peroxide was quantified using the Amplex Ultra Red detection reagent (Thermo Fisher, USA) according to previous protocols [37], and measured at *λ*_ex_ 535 nm and *λ*_em_ 590 nm using a plate reader.

### Mass spectrometry and data analysis

Oxidative modifications on Ag85B and gas plasma-treated Ag85 were examined by high-resolution mass spectrometry (MS) coupled with liquid chromatography (LC). Proteins were digested using trypsin (Promega, Germany) at an enzyme-substrate ratio of 1:20 overnight at 37 °C, followed by vacuum centrifugation (Eppendorf, Germany). Proteins were reconstituted in 0.5% formic acid (Merck, Germany) and transferred into glass vials for data acquisition using a nano system (Dionex UltiMate 3000 RSLC) connected to an Orbitrap Exploris 480 mass spectrometer with Nanospray Flex ion sources (both Thermo Scientific, USA). The trap column and PepMap C18 reversed-phase analytical column were kept at a temperature of 40 °C, and buffer A (0.1% acetic acid; Merck, Germany) and buffer B (95:5 ACN:0.1% acetic acid; Th Geyer, Germany) were used. For peptide elution, a flow rate of 300 nL/min with a gradient of 4-40% buffer B over 50 min was set. A positive polarity mode with a capillary temperature of 250 °C and a spray voltage of 2 kV was chosen for mass spectrometry analysis. Acquisition was performed in data-dependent acquisition mode (Top 15), and peptides were analyzed with a full scan (350-1200 m/z, R = 120,000 at 200 m/z) with a target of 5 × 10^3^ ions. This was followed by 15 data-dependent MS/MS scans with higher energy collision dissociation (HCD) at a maximum injection time of 50 ms, an isolation width of 1.0 m/z, and a normalized collision energy (NCE) of 30%, which were carried out in the Orbitrap (R = 15,000 were detected at 200 m/z). Proteom Discoverer with Byonic software (Proteinmetrics, USA) plug‐in was used with an in‐house-designed database to detect posttranslational modifications (Table S1). Data was filtered by byonic score, a primary indicator for PSM correctness. This score provides insight into the quality of the peptide-spectrum match. Scores above 300 are annotated as good, while scores above 400 are validated as very good. DeltaMod is the second score used to localize the oxPTM confidently. This score assesses the accuracy of oxPTM localization. Scores above 10 indicate a high likelihood that all modification placements are correct. Lastly, the Log Probability (log p-value) of the PSM is used to filter and ensure correct oxPTM calling [38]. Raw data of peptide spectrum matches (PSMs) were exported and filtered. Modifications were considered valid if a protein modification occurred in ≥66% replicates of a group.

### Animal experiment

Animal experiments were approved by the committee of the State Office of Agriculture, Food Safety and Fishery in Mecklenburg–Western Pomerania (LALLF M − V) under registration number LALLF 7221.3-2-041/17. Specific-pathogen-free, 5-7 weeks old female C57BL/6 were purchased from Charles River Laboratories (Germany). Experiments were performed at the animal facilities of the Friedrich-Loeffler-Institute (Greifswald-Riems) at 20 to 22 °C and a relative humidity of 45 ± 10% on a 12-h light/dark cycle. Animals were fed with commercial rodent chow and provided with water ad libitum. A standardized method for vaccine and boost injection, as well as daily monitoring, was used. As part of the randomization and blinding process, the animals were numbered, and immunization conditions were assigned in an alternating manner; consequently, animals receiving different treatments (Sham, Ag85B, oxAg85B) were kept together in cages. For immunization, Ag85 protein was diluted in PBS (500 μg/ml) and treated with argon gas plasma for 10 s. Anesthetized animals (isofluorane) received intramuscularly (i.m.) either 20 μl PBS (sham group; n = 5) or 20 μl PBS containing 10 μg Ag85B (n = 5) or oxAg85B (n = 5), respectively. At the indicated time points, submandibular bleeding (100-200 μl volume) under anesthesia (isofluorane) was performed for serum collection. For this, blood was collected into capillary blood collection tubes (Sarstedt, Germany) and centrifuged at 10,000 × g for 5min at room temperature. Serum was transferred into a 1.5 ml Eppendorf tube and stored at −80 °C until further usage.

Single-cell suspensions were generated from spleens by mechanical dissociation through a 70 μm cell strainer followed by erythrocyte lysis. Cells were resuspended in complete RPMI medium (RPMI supplemented with 10 % FCS and penicillin/streptomycin) and counted using a TC20 automated cell counter (Bio-Rad Laboratories, USA). For in vitro stimulation, 3 × 10^6^ splenocytes per well were cultured in complete RPMI and stimulated overnight (14–16 h) at 37 °C and 5 % CO_2_ with either Ag85B peptide pool (2 μg/ml; peptides & elephants) or Cell Activation Cocktail (BioLegend, The Netherlands). Brefeldin A (BioLegend, The Netherlands) was added during the final 4 h of incubation. For flow cytometric analysis, cells were stained with Zombie UV viability dye (BioLegend, The Netherlands) and blocked with TruStain FcX (BioLegend, The Netherlands) prior to surface staining with monoclonal antibodies against CD45, CD3, CD4, CD8α, and TCRγδ. Cells were subsequently fixed and permeabilized using the FoxP3/transcription factor staining buffer set (ThermoFisher Scientific, USA) and stained intracellularly for IFN-γ, IL-17A, granzyme B, and perforin. Finally, cells were fixed with 4 % paraformaldehyde (PFA) and acquired on a BD LSR Fortessa Cell Analyzer (BD Biosciences). Data were analyzed using BD FACSDiva and FlowJo software (version 10.5.3).

### oxAg85 ELISA

Nunc MaxiSorp plates (BioLegend, The Netherlands) were used to set up an oxAg85B ELISA. Briefly, Ag85B protein (1 mg/ml) was diluted in coating buffer (BioLegend, The Netherlands), and 100 μl protein suspensions were treated with argon gas plasma for 10 s. After overnight incubation at 4 °C, coating buffer was removed, and plates were washed three times with PBS containing 0.05% Tween 20. Blocking buffer (1% BSA in PBS) was added to each well and incubated for 30 min at RT. Diluted samples (1:20 in blocking buffer) were added to the wells, and a standard curve was prepared with anti-Ag85B antibodies (antibodies-online; Germany). Samples and standards were incubated for 2 h at RT, followed by three washing steps. Secondary antibodies (anti-mouse; Santa Cruz Biotechnology, Germany) were diluted in blocking buffer and given to the wells for 1h. Plates were washed three times with washing buffer, and anti-HRP (BioLegend, The Netherlands) antibodies were added. The plate was incubated for 30 min at RT. After washing three times, freshly mixed TMB substrate solution (BioLegend, The Netherlands) was added, and the reaction was stopped by adding 2 N H_2_SO_4_, prior to measuring absorbance at 450 nm after 5 min using a microplate reader (M200 Infinite Pro; Tecan, Switzerland).

### Splenocyte isolation and in vitro restimulation and characterization

Spleens were isolated from P25 mouse spleens or wild-type C57BL6 mice, and single-cell suspensions of splenocytes were isolated as per manufacturer's instructions (Miltenyi Biotech, Germany). For T cell experiments, 5 × 10^5^ splenocytes in fully supplemented RPMI culture medium were seeded into each well of 96-well round-bottom plates (Sarstedt, Germany), and 10 μl PBS or PBS containing Ag85B, oxAg85B I, or oxAg85B II was added. Antigen-presenting cells were cultured on a 24-well plate (2.5 × 10^6^ cells per well) and stimulated with a final concentration of 1 μg/ml proteins or oxidized AG85B, respectively. After 24 h, supernatants were collected and frozen for secretion analysis, and splenocytes were collected in V-bottom plates (Thermo Fisher Scientific, USA) for antibody staining and flow cytometric analysis. Antigen-presenting cells were scratched (cell scraper; Sarstedt, Germany) and transferred to a v-Bottom plate. Briefly, cells were washed three times with cold FACS washing buffer (Miltenyi Biotec, Germany) and stained with activated caspase 3/7 detection reagent (Thermo Fisher Scientific, USA) for live/dead discrimination and mouse Fc-block (BioLegend, The Netherlands). After incubation at room temperature for 10 min, cells for T cell analysis were stained for 30 min on ice after the addition of fluorescently conjugated monoclonal antibodies targeting CD62L (PE‐Dazzle, clone MEL‐14), CD44 (PerCP‐Cy5.5, clone IM7), CD4 (PE‐Cy7, clone L3T4), CD25 (APC, clone PC61), CD3 (Alexa Fluor 700, clone 17A2), CD69 (brilliant violet 421, clone H1.2F3), and CD8 (brilliant violet 510, clone 53–6.7). For T-cell analysis, unwanted cells were gated out using a dump channel containing zombie‐NIR as well as CD45R (APC-Fire 750, clone RA3-6B2) and I‐A/I‐E (APC-Fire 750, clone M5/114.15.2) (all BioLegend, The Netherlands). Antigen-presenting cells were stained after Fc-block and caspasee 3/7 with master mix containing CD80 (BV650, clone 16-10A1), F4/80 (PE, cloneM8), CD11b (PE-Dazzle, clone M1/70), CD86 (PE-Cy7, clone PO3), CD64 (APC, clone X54‐5/7.1), CD45R (APC-Fire 750, clone RA3-6B2), Ly6G (APC-Fire 750, clone 1A8), IAIE (AF700, clone M5/114.15.2), CD24 (BV421, clone M1/69), CD11c (BV605, clone N418) (all BioLegend, The Netherlands). After staining, cells were washed three times, and analysis was performed using flow cytometry (CytoFLEX LX; Beckman-Coulter, Germany). Data analysis was conducted using Kaluza analysis software 2.4 (Beckman‐Coulter, USA). For proliferation experiment, isolated splenocytes from P25 mice were labeled with carboxyfluorescein succinimidyl ester (CFSE, 2.5 μM; BioLegend, The Netherlands) prior to adding Ag85B or oxidized protein variants. Three days later, cells were collected and flow cytometry experiments were performed as described above.

### Secretion analysis

Frozen supernatants of stimulated cells and murine blood serum were investigated by bead‐based multiplex sandwich immunoassay according to the manufacturer's instructions (LegendPlex; BioLegend, Netherlands). Biological replicates of each group were pooled and measured in four technical replicates using flow cytometry (CytoFLEX S; Beckman‐Coulter, Germany). Data analysis software (Vigene Tech, USA) was utilized for quantification. A standard curve was calculated with attention to the analytes' specific detection limits.

### Statistical analysis

Prism 10.6 (GraphPad Software, USA) was used for graphing and statistical analysis. Correlation was conducted by Pearson's correlation. Statistical comparison of two groups was done using Mann-Whitney test. Statistical comparison of more than two groups was achieved using one-way analysis of variance (anova). Statistical comparison of more than two groups across different data sets was performed using two-way anova. Levels of significance are indicated as follows: α = 0.05 (∗), α = 0.01 (∗∗), α = 0.001 (∗∗∗) (Table S2).
