# Supplementary figures and images for: Oxidative modifications of tuberculosis antigen Ag85B alter its T cell antigenicity
Source: Redox Biol. 2026 May 28;94:104233. doi: 10.1016/j.redox.2026.104233 (PMC13233566; doi:10.1016/j.redox.2026.104233)

## Figure S1


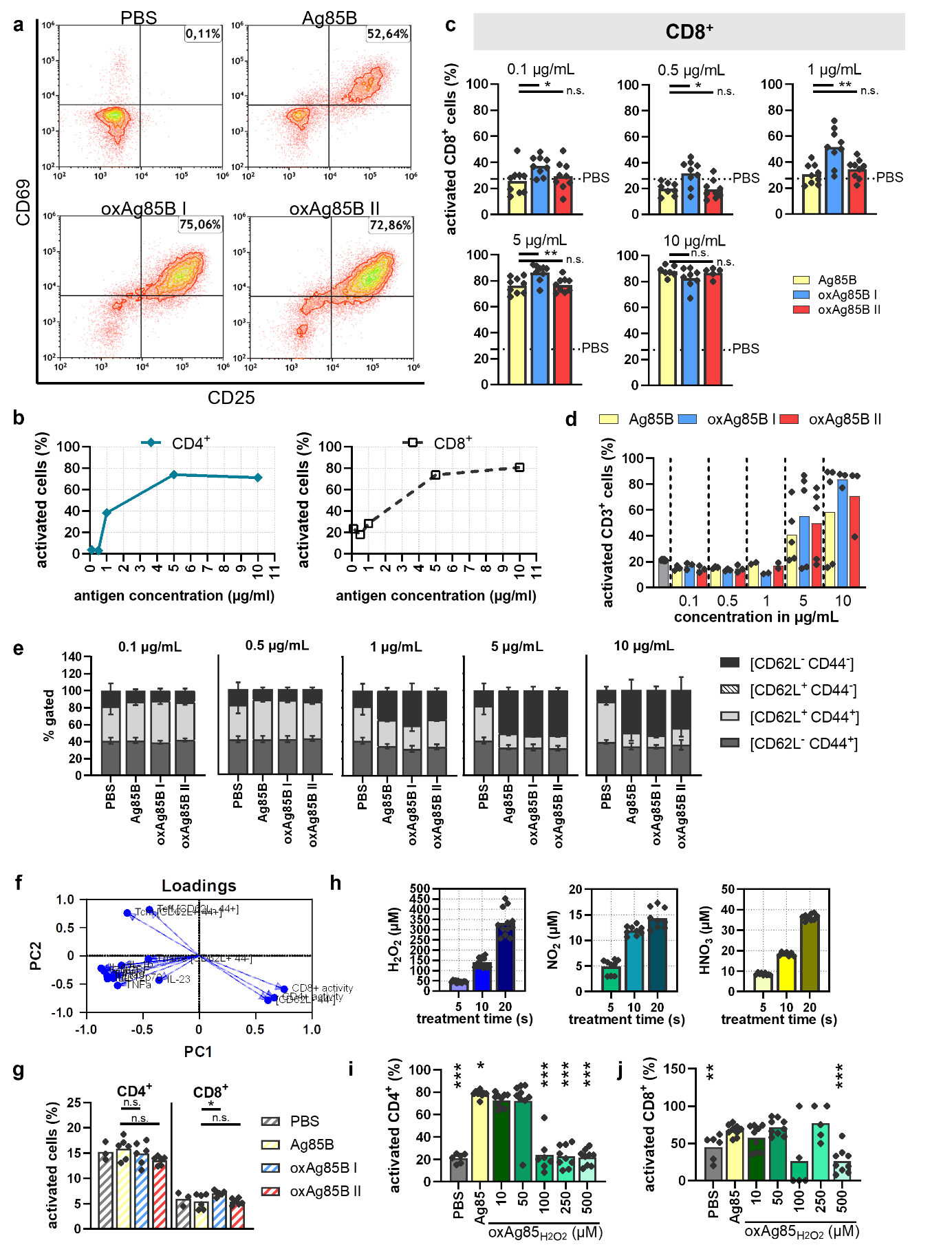


## Figure S2


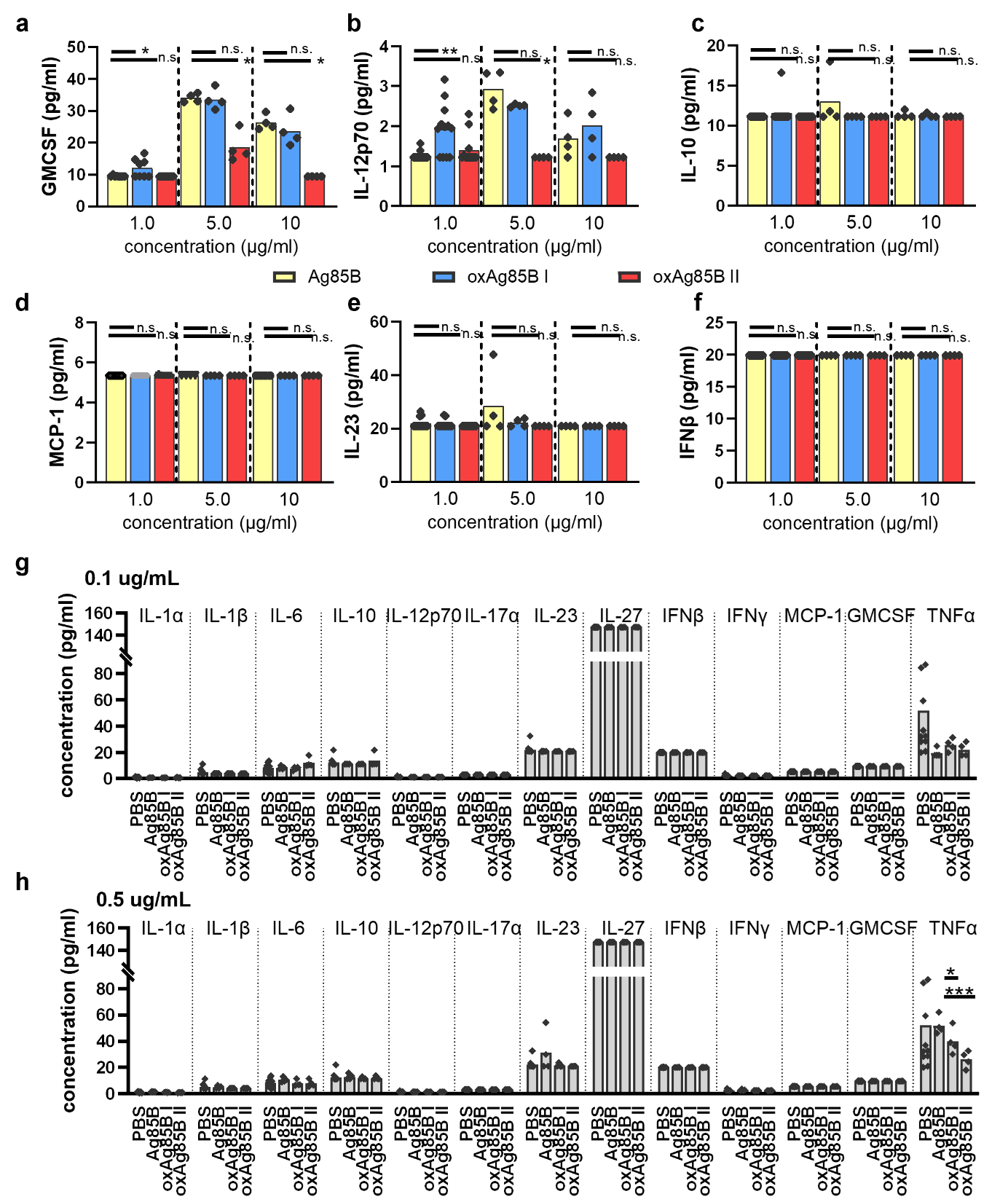


## Figure S3


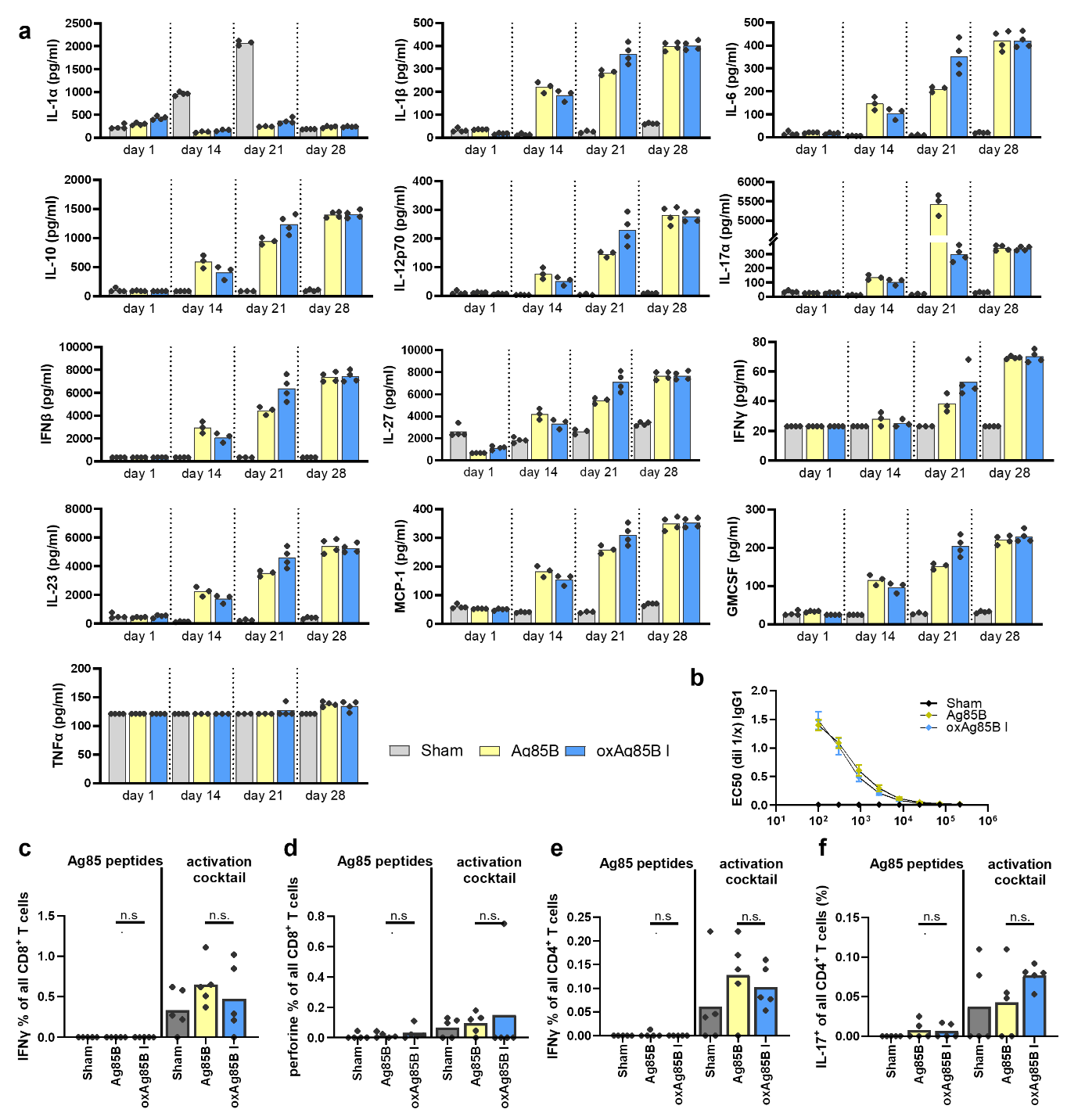


## Figure S4


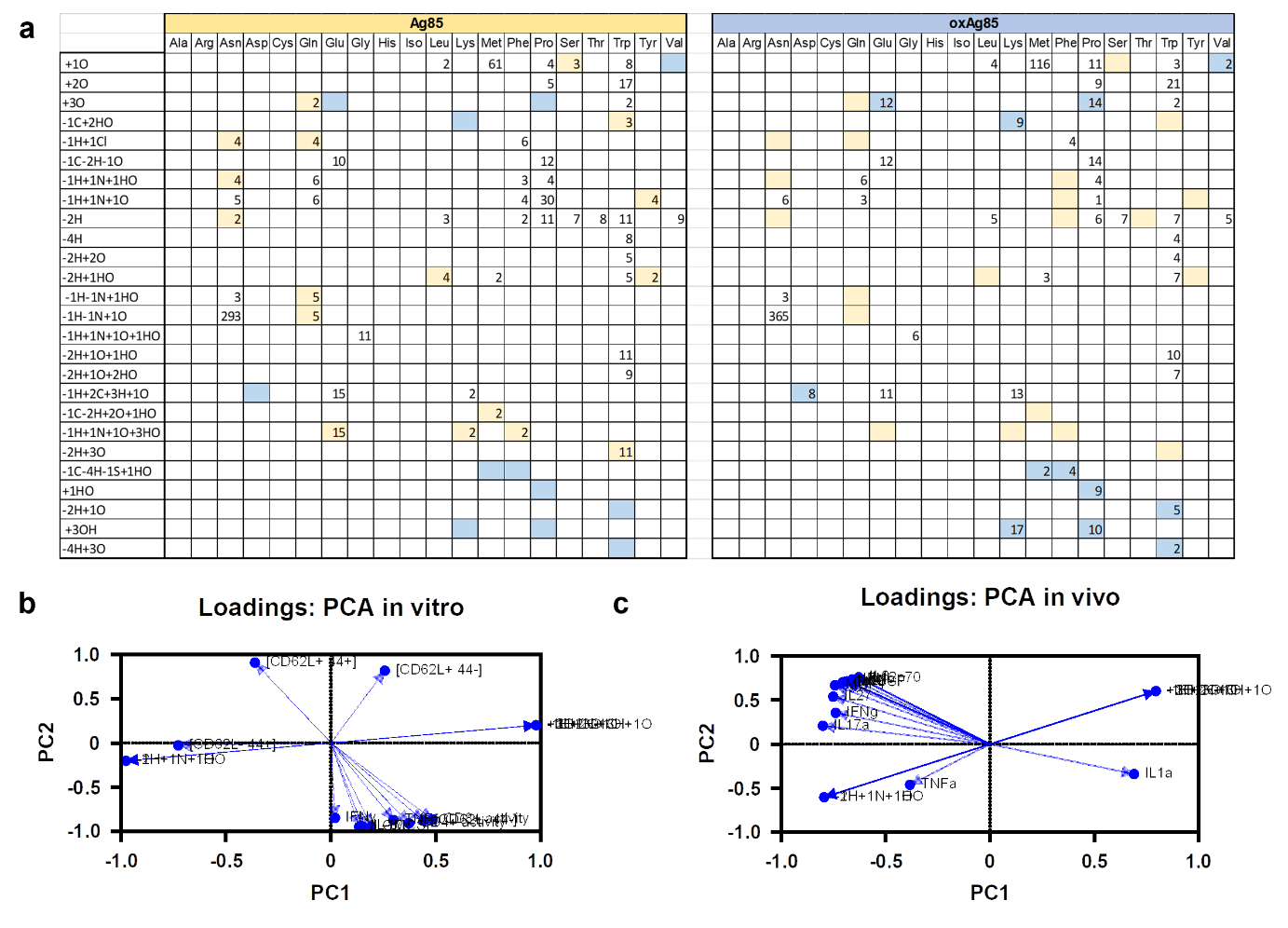


## Figure S5


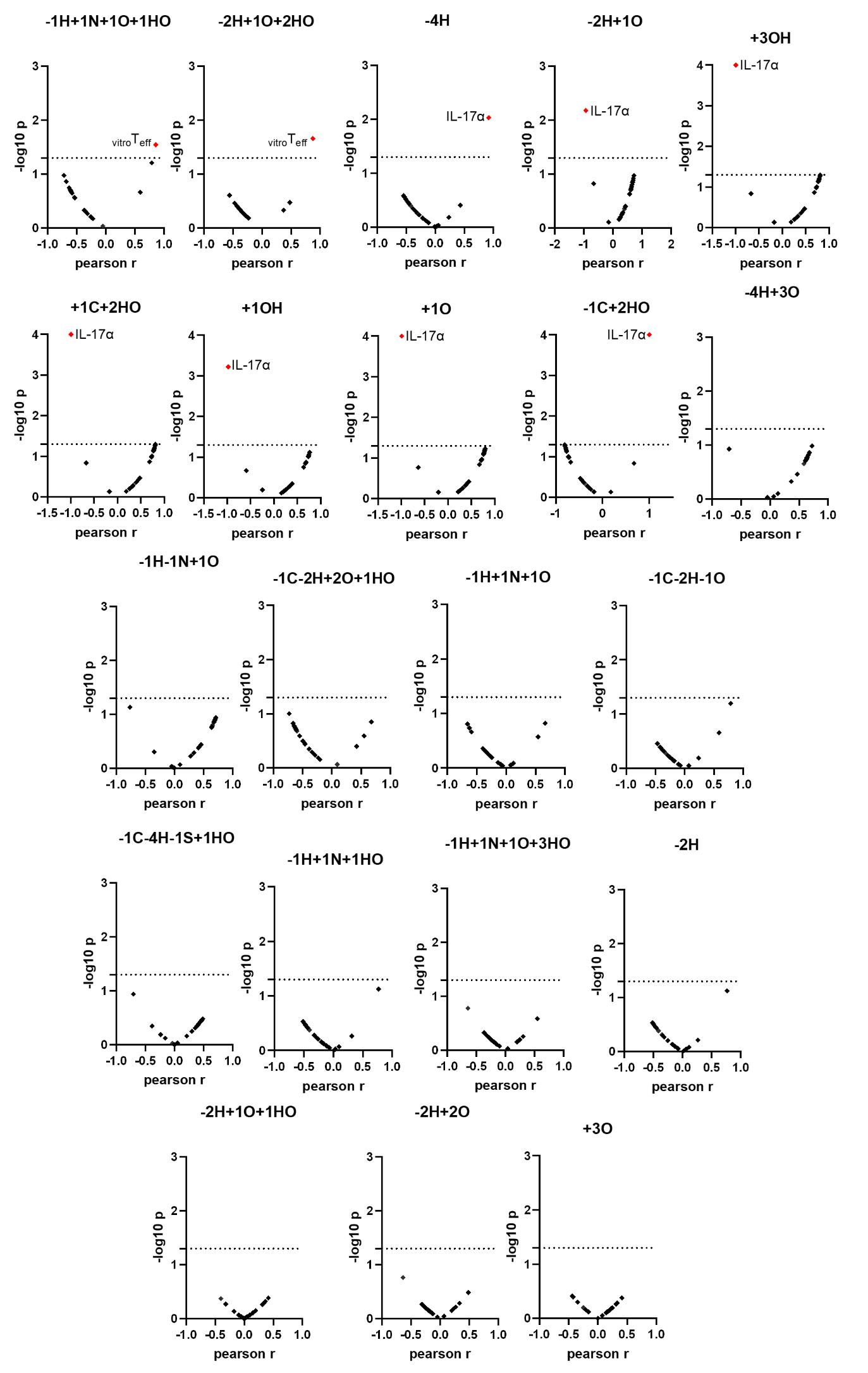

Supplement: Multimedia component 3 [file mmc3.docx]
